# Supplementary material for: Winter outdoor thermal comfort of older people with different degrees of frailty: A study in Nanyang city, China’s hot-summer and cold-winter region
Source: PLoS One. 2026 Jun 3;21(6):e0350730. doi: 10.1371/journal.pone.0350730 (PMC13232824; doi:10.1371/journal.pone.0350730)
Supplement: Attachment — Submitted filename: Response to Editor and Reviewers (1).docx [file pone.0350730.s004.docx]

**Response to Reviewers**
**Manuscript ID:** PONE-D-25-66927
**Title:** Winter outdoor thermal comfort of older people with different degrees of frailty: a study in Nanyang city, China

**Dear Dr. Editor and Reviewers,**

We would like to thank you for your valuable time and for the constructive comments regarding our manuscript. These insightful suggestions have significantly helped us improve the quality of our work.

We have carefully revised the manuscript to address all the points raised. To facilitate your review, all changes have been highlighted in the revised manuscript using red highlighting feature. Below, we provide a point-by-point response to each comment.

We hope that the revised version is now suitable for publication in PLOS One. Please do not hesitate to contact us if further modifications are required.

Thank you for your consideration.

Sincerely,

Biao WANG
Corresponding Author
Gold Mantis School of Architecture, Soochow University

**Responses to the Academic Editor**

**Comment 1 (Editor):** Please ensure that your manuscript meets PLOS ONE's style requirements, including those for file naming. The PLOS ONE style templates can be found at <https://journals.plos.org/plosone/s/file?id=wjVg/PLOSOne_formatting_sample_main_body.pdf> and [https://journals.plos.org/plosone/s/file?id=ba62/PLOSOne_formatting_sample_title_authors_affiliations.pdf](https://track.editorialmanager.com/CL0/https:%2F%2Fjournals.plos.org%2Fplosone%2Fs%2Ffile%3Fid=ba62%2FPLOSOne_formatting_sample_title_authors_affiliations.pdf/1/010f019c98c3dcf1-4cbcaada-ac05-4ad6-8dd2-2b623b792cb5-000000/Wt4PBJOxRv4HyDB2Wfp6MapfEoDo4czy5Q5CsvGW50w=252)
**Response:** Thank you for reminding us of the formatting requirements for PLOS ONE. We have carefully reviewed the provided style templates and confirmed that our revised manuscript has been formatted accordingly, including the layout of tables, text fonts, and citation formats. We are happy to make any further adjustments if necessary.

**Comment 2 (Editor):** Thank you for stating the following financial disclosure:

[This research was funded by the Science and technology project of Henan Province (252102320315). Housing and Urban Rural Construction Science and Technology Plan Project (HNJS-2024-R1), and Henan Province soft science project (242400410262, 252400410248).]

Please state what role the funders took in the study. If the funders had no role,please state: "The funders had no role in study design, data collection and analysis, decision to publish, or preparation of the manuscript."

If this statement is not correct you must amend it as needed.

Please include this amended Role of Funder statement in your cover letter; we will change the online submission form on your behalf.
**Response:** Thank you for pointing this out. We have included this amended Role of Funder statement in the cover letter as requested.

**Comment 3 (Editor):** Thank you for stating the following in your manuscript:

[This research was funded by the Science and technology project of Henan Province (252102320315). Housing and Urban Rural Construction Science and Technology Plan Project (HNJS-2024-R1), and Henan Province soft science project (242400410262, 252400410248).]

We note that you have provided funding information that is currently declared in your Funding Statement. However, funding information should not appear in the Acknowledgments section or other areas of your manuscript. We will only publish funding information present in the Funding Statement section of the online submission form.

Please remove any funding-related text from the manuscript and let us know how you would like to update your Funding Statement. Currently, your Funding Statement reads as follows:

[This research was funded by the Science and technology project of Henan Province (252102320315). Housing and Urban Rural Construction Science and Technology Plan Project (HNJS-2024-R1), and Henan Province soft science project (242400410262, 252400410248).]

Please include your amended statements within your cover letter; we will change the online submission form on your behalf.

**Response:**Thank you for your clarification. We have removed all funding related text from the manuscript and included the revised sponsor role statement in the cover letter as requested.

**Comment 4 (Editor):** We note that your Data Availability Statement is currently as follows: [All relevant data are within the manuscript and its Supporting Information files.]

Please confirm at this time whether or not your submission contains all raw data required to replicate the results of your study. Authors must share the “minimal data set” for their submission. PLOS defines the minimal data set to consist of the data required to replicate all study findings reported in the article, as well as related metadata and methods ([https://journals.plos.org/plosone/s/data-availability#loc-minimal-data-set-definition)](https://journals.plos.org/plosone/s/data-availability#loc-minimal-data-set-definition)" \t "_blank).

For example, authors should submit the following data:

- The values behind the means, standard deviations and other measures reported;
- The values used to build graphs;
- The points extracted from images for analysis.

Authors do not need to submit their entire data set if only a portion of the data was used in the reported study.

If your submission does not contain these data, please either upload them as Supporting Information files or deposit them to a stable, public repository and provide us with the relevant URLs, DOIs, or accession numbers. For a list of recommended repositories, please see [https://journals.plos.org/plosone/s/recommended-repositories](https://journals.plos.org/plosone/s/recommended-repositories).

If there are ethical or legal restrictions on sharing a de-identified data set, please explain them in detail (e.g., data contain potentially sensitive information, data are owned by a third-party organization, etc.) and who has imposed them (e.g., an ethics committee). Please also provide contact information for a data access committee, ethics committee, or other institutional body to which data requests may be sent. If data are owned by a third party, please indicate how others may request data access.

**Response:** Thank you for the reminder. We have now uploaded the raw data as Supporting Information files. The updated Data Availability Statement reads: "All relevant data are within the manuscript and its Supporting Information files."

**Comment 5 (Editor):** Your ethics statement should only appear in the Methods section of your manuscript. If your ethics statement is written in any section besides the Methods, please move it to the Methods section and delete it from any other section. Please ensure that your ethics statement is included in your manuscript, as the ethics statement entered into the online submission form will not be published alongside your manuscript.

**Response:** Thank you for your guidance. We have made the required revisions, and the ethical statement is only located in the methods section of our manuscript and does not appear elsewhere.

**Comment 6 (Editor):** We note that Figure 1 in your submission contains images which may be copyrighted. All PLOS content is published under the Creative Commons Attribution License (CC BY 4.0), which means that the manuscript, images, and Supporting Information files will be freely available online, and any third party is permitted to access, download, copy, distribute, and use these materials in any way, even commercially, with proper attribution. For more information, see our copyright guidelines: [http://journals.plos.org/plosone/s/licenses-and-copyright](http://journals.plos.org/plosone/s/licenses-and-copyright).

We require you to either (1) present written permission from the copyright holder to publish these figures specifically under the CC BY 4.0 license, or (2) remove the figures from your submission:

1. You may seek permission from the original copyright holder of Figure 1 to publish the content specifically under the CC BY 4.0 license.

We recommend that you contact the original copyright holder with the Content Permission Form ([http://journals.plos.org/plosone/s/file?id=7c09/content-permission-form.pdf)](http://journals.plos.org/plosone/s/file?id=7c09/content-permission-form.pdf)) and the following text:

“I request permission for the open-access journal PLOS ONE to publish XXX under the Creative Commons Attribution License (CCAL) CC BY 4.0 ([http://creativecommons.org/licenses/by/4.0/)](http://creativecommons.org/licenses/by/4.0/)). Please be aware that this license allows unrestricted use and distribution, even commercially, by third parties. Please reply and provide explicit written permission to publish XXX under a CC BY license and complete the attached form.”

Please upload the completed Content Permission Form or other proof of granted permissions as an "Other" file with your submission.

In the figure caption of the copyrighted figure, please include the following text: “Reprinted from [ref] under a CC BY license, with permission from [name of publisher], original copyright [original copyright year].”

2. If you are unable to obtain permission from the original copyright holder to publish these figures under the CC BY 4.0 license or if the copyright holder’s requirements are incompatible with the CC BY 4.0 license, please either i) remove the figure or ii) supply a replacement figure that complies with the CC BY 4.0 license. Please check copyright information on all replacement figures and update the figure caption with source information. If applicable, please specify in the figure caption text when a figure is similar but not identical to the original image and is therefore for illustrative purposes only.

**Response:** Thank you for your guidance. To ensure full compliance with the CC BY 4.0 license, we have removed the maps inside.

**Comment 7 (Editor):** We note that Figure 1 in your submission contains map and satellite images which may be copyrighted. All PLOS content is published under the Creative Commons Attribution License (CC BY 4.0), which means that the manuscript, images, and Supporting Information files will be freely available online, and any third party is permitted to access, download, copy, distribute, and use these materials in any way, even commercially, with proper attribution. For these reasons, we cannot publish previously copyrighted maps or satellite images created using proprietary data, such as Google software (Google Maps, Street View, and Earth). For more information, see our copyright guidelines: [http://journals.plos.org/plosone/s/licenses-and-copyright](http://journals.plos.org/plosone/s/licenses-and-copyright).

We require you to either (1) present written permission from the copyright holder to publish these figures specifically under the CC BY 4.0 license, or (2) remove the figures from your submission:

1. You may seek permission from the original copyright holder of Figure 1 to publish the content specifically under the CC BY 4.0 license.

We recommend that you contact the original copyright holder with the Content Permission Form ([http://journals.plos.org/plosone/s/file?id=7c09/content-permission-form.pdf)](http://journals.plos.org/plosone/s/file?id=7c09/content-permission-form.pdf)) and the following text:

“I request permission for the open-access journal PLOS ONE to publish XXX under the Creative Commons Attribution License (CCAL) CC BY 4.0 ([http://creativecommons.org/licenses/by/4.0/)](http://creativecommons.org/licenses/by/4.0/)). Please be aware that this license allows unrestricted use and distribution, even commercially, by third parties. Please reply and provide explicit written permission to publish XXX under a CC BY license and complete the attached form.”

Please upload the completed Content Permission Form or other proof of granted permissions as an "Other" file with your submission.

In the figure caption of the copyrighted figure, please include the following text: “Reprinted from [ref] under a CC BY license, with permission from [name of publisher], original copyright [original copyright year].”

2. If you are unable to obtain permission from the original copyright holder to publish these figures under the CC BY 4.0 license or if the copyright holder’s requirements are incompatible with the CC BY 4.0 license, please either i) remove the figure or ii) supply a replacement figure that complies with the CC BY 4.0 license. Please check copyright information on all replacement figures and update the figure caption with source information. If applicable, please specify in the figure caption text when a figure is similar but not identical to the original image and is therefore for illustrative purposes only.

The following resources for replacing copyrighted map figures may be helpful:

USGS National Map Viewer (public domain): [http://viewer.nationalmap.gov/viewer/](http://viewer.nationalmap.gov/viewer/)
The Gateway to Astronaut Photography of Earth (public domain): [http://eol.jsc.nasa.gov/sseop/clickmap/](http://eol.jsc.nasa.gov/sseop/clickmap/), Maps at the CIA (public domain): [https://www.cia.gov/library/publications/the-world-factbook/index.html](https://www.cia.gov/library/publications/the-world-factbook/index.html) and [https://www.cia.gov/library/publications/cia-maps-publications/index.html](https://track.editorialmanager.com/CL0/https:%2F%2Fwww.cia.gov%2Flibrary%2Fpublications%2Fcia-maps-publications%2Findex.html/1/010f019c98c3dcf1-4cbcaada-ac05-4ad6-8dd2-2b623b792cb5-000000/99D7xSXrXJzvVKLUjJGJ2dLgmScnJXJCcb7eu-VyeVs=252), NASA Earth Observatory (public domain): [http://earthobservatory.nasa.gov/](http://earthobservatory.nasa.gov/), Landsat: [http://landsat.visibleearth.nasa.gov/](http://landsat.visibleearth.nasa.gov/), USGS EROS (Earth Resources Observatory and Science (EROS) Center) (public domain): [http://eros.usgs.gov/#](http://eros.usgs.gov/), Natural Earth (public domain): [http://www.naturalearthdata.com/](http://track.editorialmanager.com/CL0/http:%2F%2Fwww.naturalearthdata.com%2F/1/010f019c98c3dcf1-4cbcaada-ac05-4ad6-8dd2-2b623b792cb5-000000/hbjqsnkjrQrGWb8u44bECA0Q3N9J8d-2t38lFQ325lM=252)

**Response:** Thank you for your detailed guidance regarding Figure 1. To ensure full compliance with the CC BY 4.0 license, we have removed the maps inside.

**Comment 8 (Editor):** Please ensure that you refer to Figure 6 in your text as, if accepted, production will need this reference to link the reader to the Figures.

**Response:** Thank you for the reminder. We have ensured that Figure 6 is appropriately cited in the main text to guide readers.

**Comment 9(Editor):** We note you have included a table to which you do not refer in the text of your manuscript. Please ensure that you refer to Table 2 in your text; if accepted, production will need this reference to link the reader to the Tables.

**Response:** Thank you for pointing this out. We have now added a citation to Table 2 in the main text (in section 2.2). The table is now properly referenced to guide readers.

**Responses to Reviewer 1**

**Comment 1:**  In the Introduction, the authors need to more clearly emphasize how frailty-based classification differs from traditional age-based grouping and why it provides added scientific value for outdoor thermal comfort research.
**Response:** Thank you for this important comment. We have revised the Introduction. The revised text now reads:

*However, age based grouping is essentially a division based on the "time dimension", which assumes homogeneity of physical function among the elderly within the same age group. However, age itself cannot accurately reflect the true degree of physiological decline (i.e. weakness) of the elderly. In contrast, classification based on the degree of weakness is a classification based on the "health dimension". It directly focuses on the differences in stress resistance among the elderly due to decreased physiological reserves and multiple system dysfunction. The scientific value of this classification method lies in its breaking away from the traditional "one size fits all" research perspective of age grouping, and recognizing and quantifying the heterogeneity of the elderly population. In fact, there are significant differences in physiological regulation abilities (such as sweating, vasoconstriction) and psychological perception thresholds among elderly people with different degrees of weakness (such as strong, pre weak, and weak), leading to their different responses to changes in outdoor environments. For example, research on indoor spaces in residential buildings in hot summers and cold winters in China has shown that elderly people with different degrees of frailty exhibit different thermal adaptation behaviors in winter; Similarly, in the case of sudden changes in indoor temperature during summer, there are significant differences in subjective feelings and physiological regulation among elderly people with different degrees of weakness.*

*Therefore, introducing the concept of "frailty level" into outdoor thermal comfort research has additional scientific value in that it goes beyond the rough division based solely on the time dimension (age) and instead starts from the actual physiological health status of the human body, providing a more explanatory theoretical framework for explaining and predicting the huge individual thermal comfort differences in the elderly population. This will help to develop more refined and inclusive design standards for aging friendly outdoor environments in the future, rather than just serving "statistically significant elderly people".*

This revision appears on Lines 112-138.

**Comment 2:**  In Section 2.3, please briefly explain how the FRAIL questionnaire was administered.

**Response:** Thank you for the suggestion. We have added a brief explanation of the FRAIL questionnaire administration in Section 2.3. The revised text now reads:

*"The FRAIL scale was administered through face-to-face interviews conducted simultaneously with the thermal comfort survey. Each participant was asked the five FRAIL questions verbally by the investigator, and responses were recorded immediately to ensure accuracy and completeness."*

This addition appears on Lines 226-229.

**Comment 3:** In Section 2.4, please specify the assumed values or ranges of the key personal parameters used in the RayMan software.

**Response:** Thank you for the comment. We have revised Section 2.4 to specify the assumed values for key personal parameters used in the RayMan software. The updated text now reads:

*The required input parameters included meteorological data (air temperature, relative humidity, wind speed, and mean radiant temperature derived from global radiation) and personal parameters. Based on the surveyed population characteristics, the following standard assumptions were applied: height (1.65 m, representing the average of the older adult sample), weight (65 kg, based on measured sample average), age (72 years, sample mean), gender (male/female as recorded), clothing insulation (0.9 clo, typical value for the season and survey period).*

This addition appears on Lines 255-261.

**Comment 4:** In the Results section, please specify the type of regression model used and report the relevant statistical indicators. A brief explanation of how the neutral temperature (NPET) was derived from the regression results would help improve clarity.

**Response:** Thank you for your valuable comment. We have revised Section 4 (Results) to specify the regression model type and provide a clearer explanation of how neutral temperature was derived. The updated text now reads:

*The regression results showed good model fit for all three groups: non-frail (R² = 0.67, p = 0.188), pre-frail (R² = 0.82, p = 0.076), and frail (R² = 0.87, p < 0.049).*

This addition appears on Lines 326-328.

*The MTSV was calculated for each PET interval, and a univariate linear regression model was fitted between PET (independent variable) and MTSV (dependent variable). The regression results showed strong linear relationships for all three groups: non-frail (R² = 0.85, p < 0.001), pre-frail (R² = 0.87, p < 0.001), and frail (R² = 0.91, p < 0.001). The regression equations are presented in Fig 7. The neutral temperature (NPET), defined as the PET value at which MTSV = 0, was derived by solving the regression equation for MTSV = 0, yielding NPET. The calculated NPET values were 10.29°C for the non-frail group, 9.60°C for the pre-frail group, and 8.65°C for the frail group，indicating that NPET decreases as frailty severity increases.*

This addition appears on Lines 365-373.

*The unacceptability rate (proportion of votes indicating 'unacceptable') was calculated for each interval, and a binary logistic regression model was fitted with PET as the independent variable and thermal acceptability as the dependent variable. The results are shown in Fig 8. The regression results showed good model fit for all three groups: non-frail (R² = 0.94, p < 0.001), pre-frail (R² = 0.92, p < 0.001), and frail (R² = 0.94, p < 0.001).*

This addition appears on Lines 391-396.

**Comment 5:** The finding in the Discussion that thermal sensitivity decreases with increasing frailty is interesting. However, The Discussion could benefit from a more explicit physiological or behavioral explanation. Please link the findings more clearly to existing indoor or medical studies on frailty and thermal perception.

**Response:** Thank you for this insightful comment. We agree that providing explicit physiological and behavioral explanations strengthens the Discussion. The updated text now reads:

*This study found that as the degree of frailty increases, the outdoor thermal sensitivity of older people gradually decreases. This reduced sensitivity can be attributed to physiological and behavioral mechanisms associated with frailty. Physiologically, frailty involves age-related declines in autonomic nervous system function, including impaired vasoconstriction and reduced sweating capacity, which blunt the body's ability to detect and respond to thermal stimuli[48,71]. Behaviorally, frail older adults may have reduced activity levels and spend less time outdoors, potentially leading to diminished thermal perception through reduced environmental exposure and adaptation[72].*

*Our finding aligns with studies on older adults with varying frailty levels in China's hot-humid regions during summer[48], which also reported blunted thermal perception in frail individuals. However, it contrasts with a study on extreme high-temperature weather outdoors in Beijing[73], which found that chronic disease patients had higher neutral temperatures than healthy individuals. This discrepancy may be explained by differences in health status definitions—chronic disease does not necessarily equate to frailty, as frailty encompasses multidimensional physiological decline beyond specific disease diagnoses[74]. Additionally, the Beijing study used UTCI rather than PET, and these indices incorporate different thermophysiological assumptions, which may affect sensitivity estimates.*

*Interestingly, an indoor study of frail older adults in China's hot summer and cold winter region found that they were more sensitive to heat and had a narrower comfortable temperature range[49]—the opposite of our outdoor findings. This divergence highlights the critical role of environmental context: indoors, frail individuals are exposed to stable, controlled conditions where their impaired thermoregulatory systems may struggle to maintain comfort; outdoors, they face dynamic environmental stimuli that may exceed their perceptual thresholds, resulting in blunted sensation. Furthermore, frail older adults may be less capable of taking timely adaptive actions (e.g., seeking shade, adjusting clothing) when outdoors, making them more vulnerable to extreme temperatures despite reporting lower sensitivity[48].*

*The above differences may also stem from methodological factors, including different frailty assessment tools (this study used the FRAIL scale; Zhou et al.[49-51] used the Fried Frailty Phenotype), climate backgrounds, cultural differences in clothing insulation, and questionnaire design. Future research should systematically compare the performance of UTCI and PET in assessing outdoor thermal comfort for older adults with different frailty levels, to identify the most appropriate evaluation indicators for regions with hot summers and cold winters.*

This addition appears on Lines 638-672.

**Responses to Reviewer 2**

**Comment 1:** Abbreviations and Formatting Issues
There are inconsistencies in the formatting of the Abbreviations section. Some full terms are capitalized while others are not; the formatting should be standardized throughout. In addition, widely recognized institutional abbreviations such as ASHRAE do not need to be listed in the abbreviations table. Abbreviations should be defined only at their first occurrence in the main text and subsequently used in their abbreviated form without repeated explanation (e.g., TSV and related indices).
Some equations and numbering formats appear inconsistent and should be revised according to the journal's formatting requirements.

**Response:** Thank you for your careful review of our manuscript's formatting. We have made the following revisions to address all the issues raised:

Abbreviations section formatting: We have standardized the capitalization throughout the Abbreviations section, ensuring that all full terms follow consistent sentence case (only first word capitalized).

Removal of widely recognized abbreviations: Following your guidance, we have removed widely recognized institutional abbreviations such as ASHRAE from the Abbreviations table, as they do not require definition.

Equation and numbering format: We have revised equations according to the journal's style requirements.

All changes have been highlighted in the revised manuscript. We appreciate your attention to these details and believe the manuscript now fully complies with the journal's formatting standards.

**Comment 2:** Timeliness and Accuracy of References
Several references are relatively outdated (e.g., References 2, 3, and 6 are from around 2010). Considering the rapid development of outdoor thermal comfort research in recent years, the authors are encouraged to incorporate more recent studies (particularly from the last five years) to strengthen the manuscript's relevance and academic currency.
In addition, the journal name in Reference 2 is incorrect; it should be Ageing Research Reviews. The authors are advised to thoroughly check all references for accuracy, including journal titles, volume and issue numbers, page ranges, and formatting consistency.

**Response:**Thank you for your valuable suggestions regarding the references. We have carefully addressed both concerns as follows:

Updated references for timeliness: Following your recommendation, we have replaced several outdated references (including the original References 2, 3, and 6 from around 2010) with more recent studies published.

The updated references now reads:

*[2] P.Kumar, A.Sharma, Study on importance, procedure, and scope of outdoor thermal comfort –A review, Sustainable Cities and Society, 61(2020),102297, https://doi.org/10.1016/j.scs.2020.102297.*

*[3]S.Shooshtarian, C.K.C.Lam, I.Kenawy, Outdoor thermal comfort assessment: A review on thermal comfort research in Australia, Building and Environment, 177(2020)106917, https://doi.org/10.1016/j.buildenv.2020.106917.*

*[6] Z.Liu, J.Li, T.Xi. A Review of Thermal Comfort Evaluation and Improvement in Urban Outdoor Spaces. Buildings,13(2023)3050. https://doi.org/10.3390/buildings13123050.*

The revised reference list has been fully updated in the manuscript, and all changes are highlighted. We appreciate your careful attention to these details, which have significantly improved the quality of our manuscript.

**Comment 3:** Climate Zone Specification in the Title
The title would benefit from explicitly specifying the climate zone (i.e., hot-summer and cold-winter region, HSCW) to improve clarity and scientific precision.
**Response:**

Thank you for your suggestion. We have updated the title to explicitly specify the climate zone. The new title is:

"*Winter outdoor thermal comfort of older people with different degrees of frailty: a study in Nanyang city, China's hot-summer and cold-winter region*".

**Comment 4:** Insufficient Climatic Context in the Introduction

Furthermore, the Introduction should provide a clearer justification for selecting Nanyang as the case study city. The authors should elaborate on its climatic representativeness, urban characteristics, or research gaps in existing studies to better justify the choice of study location.

The Introduction would be strengthened by including a more detailed description of Nanyang's climatic characteristics and comparing them with other cities in the HSCW region previously studied (e.g., Changsha, Wuhan, Chengdu). Highlighting similarities and differences would help clarify how this study complements or extends existing research and better demonstrate its contribution.

**Response:** Thank you for this insightful suggestion. We have strengthened the Introduction by adding a detailed description of Nanyang's climatic characteristics and comparing it with other HSCW cities previously studied. The revised text now reads:

*Currently, most outdoor thermal comfort research focuses on popular provincial capitals, such as Changsha, Wuhan, and Chengdu, leaving prefecture-level cities like Nanyang understudied. However, these smaller cities house significant aging populations and face distinct microclimatic conditions that may differ from major metropolitan areas. Nanyang, located in southwestern Henan Province, is a representative city in China's hot-summer and cold-winter (HSCW) climate zone, characterized by humid, hot summers and cold, dry winters. Its urban morphology, population density, and aging demographics are typical of many medium-sized Chinese cities, making it an ideal case for understanding the thermal comfort needs of older adults beyond provincial capitals. Furthermore, as microclimates and individual variables jointly influence human thermal sensation, research has increasingly moved beyond mixed-population studies to examine population subgroups based on gender, age, and activity ^[36-38]^. Investigating frailty thermal responses in a representative HSCW city like Nanyang addresses a critical research gap and provides insights applicable to similar cities across the region.*

This addition appears on Lines 83-96.

**Comment 5:** Figures and Graphical Clarity
In several figures, the axis labels and legends are too small, which affects readability. The font size should be enlarged to enhance clarity. The font style used in Figure 11 is inconsistent with the other figures and should be standardized.
The authors are encouraged to carefully review all figures to ensure consistent formatting, adequate resolution, uniform font style, appropriate line thickness, and clear color contrast. If necessary, the figures should be redrawn to improve overall visual quality and professionalism.

**Response:** Thank you for your careful attention to the visual presentation of our figures. We have thoroughly revised all figures to address the readability and consistency issues:

Enlarged axis labels and legends: We have increased the font size for all axis labels, tick marks, and legends across all figures to ensure optimal readability. The minimum font size now meets the journal's requirements for print and online viewing.

Standardized Fig 11: We have reformatted Figure 11 to use the same font style (Arial), line thickness, and color scheme as the other figures, ensuring visual consistency throughout the manuscript.

Comprehensive figure review: We have systematically reviewed all figures (Figures 1-11) to ensure:

Consistent font style (Arial) and size across all elements.

Appropriate line thickness for clear visibility.

We believe these improvements enhance the visual quality and professionalism of the manuscript.

Thank you again for helping us identify these issues.
