# Supplementary material for: Winter outdoor thermal comfort of older people with different degrees of frailty: A study in Nanyang city, China’s hot-summer and cold-winter region
Source: PLoS One. 2026 Jun 3;21(6):e0350730. doi: 10.1371/journal.pone.0350730 (PMC13232824; doi:10.1371/journal.pone.0350730)
Supplement: Attachment — Submitted filename: Response to Reviewers 4.1.docx [file pone.0350730.s005.docx]

**Responses to Reviewer 1**

**Comment 1:**  In Chapter 2.2, the measurement period appears limited (6 days), and representativeness is not discussed. Whether these days represent typical winter conditions and how measurement days were selected.
**Response:** We sincerely thank the reviewer for raising this important point regarding the representativeness of our measurement period. We agree that clarifying the selection criteria and demonstrating the representativeness of the measured data are essential for the validity of the study. We have revised Section 2.2 to provide a detailed explanation of the measurement day selection and the data's representativeness.

The revised text now reads:

*The selection of these six measurement days was based on the local long-term meteorological records (2011–2021) to ensure representativeness of typical winter conditions in Nanyang City. Specifically, the selected dates cover the coldest period (January) and the transition months (December and February). According to historical data, daily average temperatures during winter typically range from -1.9°C to 8.5°C, and the recorded air temperatures during our measurement days (ranging from -2.9°C to 15.1°C) captured the full spectrum of typical winter weather, including both the lowest monthly averages.*

*All measurement days were sunny or partly cloudy, representing the most common weather conditions for outdoor activities among the elderly, as overcast or rainy days usually deter them from visiting parks. We intentionally included the Winter Solstice (December 21) as a measurement day. On this day, the sun reaches its lowest solar elevation angle of the year, which results in the weakest solar radiation intensity and the longest shadows. This represents a critical extreme condition for outdoor thermal comfort in winter, allowing us to capture the lower bound of thermal exposure that elderly individuals may experience.*

**Comment 2:**  In Chapter 3.2, the preferred PET value for the frail group is unusually high for winter outdoor conditions, which raises concerns about interpretation. Please provide an explanation in the Discussion section.

**Response:** We sincerely thank the reviewer for this insightful observation. We agree that the unusually high preferred PET value (29.0°C) for the frail group requires careful interpretation to avoid potential misunderstanding. In response, we have added a dedicated explanation in Section 4.1 of the Discussion to contextualize this finding.

The revised text now reads:

*It is also important to address the unusually high preferred PET value (29.0°C) derived for the frail group in the probit regression analysis (Section 3.2). While this value is considerably higher than the typical winter outdoor temperatures recorded in this study (ranging from -2.9°C to 15.4°C), it should be interpreted with caution. This result primarily reflects the statistical extrapolation of the probit model beyond the range of observed PET values. Since the survey was conducted exclusively in winter conditions, there were insufficient thermal sensation votes in the warm-to-hot range to constrain the right tail of the probit curve. Consequently, the estimated preferred temperature for the frail group represents a mathematical projection of their desire for warmer conditions, rather than a literal indication that they would feel comfortable at 29°C outdoors in winter.*

*Nevertheless, this finding carries meaningful implications. The substantially higher preferred PET among frail older adults, compared with the non-frail (11.9°C) and pre-frail (16.2°C) groups, suggests that frail individuals have a stronger psychological and physiological preference for warmer environments when exposed to cold winter conditions. This aligns with their reduced metabolic heat production and impaired thermoregulatory capacity, which necessitate greater external heat input to achieve thermal comfort. In practical terms, this indicates that for frail older adults, winter outdoor spaces should prioritize maximizing solar radiation exposure, providing radiant heating elements (e.g., heated seating), and offering enclosed or semi-enclosed warm shelters within urban parks. Future studies should aim to validate this finding by conducting all-season measurements that include transitional seasons (spring and autumn) or by employing controlled thermal chamber experiments with frail participants to obtain preference data across a broader PET range without relying on model extrapolation.*

**Comment 3:**  *In Chapter 3.3, the manuscript switches from ordered regression to Spearman correlation without sufficient justification.*

**Response:** We thank the reviewer for this important methodological observation. We agree that the transition from ordered regression to Spearman correlation requires clear justification to ensure methodological transparency. We have now revised Section 3.3 to provide a detailed explanation of this analytical decision.

The revised text now reads:

*This limited outcome was likely due to the relatively small sample size within each frailty subgroup and the multicollinearity among predictors, which reduced the statistical power of the multivariate model to detect the individual contributions of personal, social, and psychological factors.*

*Given these limitations, we employed univariate regression analysis using Spearman correlation as a complementary approach to explore the relationships between each independent variable and thermal perception outcomes (TSV, TCV, and TAV). Spearman correlation was selected for three reasons. First, it does not assume linearity or normality, making it robust for analyzing ordinal data such as Likert-scale thermal sensation votes. Second, it allows for the examination of individual factor contributions without the risk of overfitting due to multicollinearity. Third, it is widely used in exploratory outdoor thermal comfort studies to identify key influencing factors prior to more complex modeling. This approach enabled us to reveal how the influence of personal, social, and psychological factors varies across frailty levels.*

Thank you again for helping us identify these issues.
