# Supplementary material for: Winter outdoor thermal comfort of older people with different degrees of frailty: A study in Nanyang city, China’s hot-summer and cold-winter region
Source: PLoS One. 2026 Jun 3;21(6):e0350730. doi: 10.1371/journal.pone.0350730 (PMC13232824; doi:10.1371/journal.pone.0350730)
Supplement: Attachment — Submitted filename: Response to Reviewers5.7.docx [file pone.0350730.s006.docx]

**Response to Reviewers**
**Manuscript ID:** PONE-D-25-66927
**Title:** Winter outdoor thermal comfort of older people with different degrees of frailty: a study in Nanyang city, China

May 7, 2026

**Dear Dr. Editor and Reviewers,**

We thank the reviewer for raising this essential methodological concern. We completely agree that deriving a precise numerical preferred temperature from data far outside the observed PET range is not statistically valid or reliable. We therefore no longer claim that 29.0°C is a credible quantitative estimate of the frail group’s thermal preference. Instead, we have revised the manuscript to treat this result as only a qualitative or directional signal—namely, that frail older adults have a stronger relative preference for warmer conditions compared to non-frail and pre-frail groups—while explicitly stating that the absolute value should be disregarded.Thank you for your consideration.

Sincerely,

Biao WANG
Corresponding Author
Gold Mantis School of Architecture, Soochow University

**Responses to Reviewer #1:**

**Comment :**
The explanation attributes the higher preferred PET value (29.0 °C) to model extrapolation; however, the observed PET range falls significantly below this figure. Please clarify the validity and reliability of deriving a preferred value outside the range of the empirical data.

**Response:** We thank the reviewer for this essential methodological critique. The reviewer is absolutely correct: deriving a precise numerical “preferred temperature” from data far outside the observed PET range is neither statistically valid nor reliable. We acknowledge this limitation without reservation.

**(a) Results section (Section 3.2)**

**Original text:**

As shown in Fig 9, the preferred PET was 11.9°C for non-frail older adults, 16.2°C for pre-frail older adults, and 29.0°C for frail older adults.

**Revised text:**

As shown in Fig 9, the probit regression curves intersect at PET values of 11.9°C for the non-frail group and 16.2°C for the pre-frail group. For the frail group, the intersection occurred at 29.0°C. Importantly, this value lies far outside the range of PET values actually recorded during the winter survey. Therefore, the 29.0°C estimate is a statistical extrapolation that lacks empirical support and should not be interpreted as a realistic comfortable temperature in winter. Instead, the key finding from the probit analysis is the consistent ordering: frail > pre-frail > non-frail, indicating that frail older adults have a stronger relative preference for warmer conditions. Future studies with all-season data or controlled thermal exposures are needed to reliably quantify the absolute preferred temperature for frail individuals.

This addition appears on Lines 437-446.

**(b) Discussion section (Section 4.2、4.5)**

Section 4.2：

**Original text:**

It is also important to address the unusually high preferred PET value (29.0°C) derived for the frail group in the probit regression analysis (Section 3.2). While this value is considerably higher than the typical winter outdoor temperatures recorded in this study (ranging from -2.9°C to 15.4°C), it should be interpreted with caution. This result primarily reflects the statistical extrapolation of the probit model beyond the range of observed PET values. Since the survey was conducted exclusively in winter conditions, there were insufficient thermal sensation votes in the warm-to-hot range to constrain the right tail of the probit curve. Consequently, the estimated preferred temperature for the frail group represents a mathematical projection of their desire for warmer conditions, rather than a literal indication that they would feel comfortable at 29°C outdoors in winter.

**Revised text:**

The probit regression analysis (Section 3.2) yielded an estimated preferred PET of 29.0°C for frail older adults. However, this value is derived by extrapolating the logistic curves far beyond the observed PET range. Statistically, such extrapolation assumes that the relationship between PET and choice probability remains logistic outside the data range, an assumption that cannot be verified with the current dataset. Therefore, this 29.0°C figure should not be cited or used as a quantitative benchmark. Its only scientifically defensible use is to indicate a qualitative ordering: among the three frailty groups, frail individuals expressed the strongest desire for warmer conditions, followed by pre-frail, and then non-frail. This ordering is consistent with the neutral temperature results (NPET decreasing with frailty) and with their reduced metabolic heat production. To reliably quantify the preferred temperature of frail older adults, future research should either (a) conduct measurements across all seasons (including spring and autumn) to capture a wider PET range, or (b) use controlled thermal chamber experiments with frail participants, which can safely expose them to a broader temperature spectrum without relying on statistical extrapolation.

This addition appears on Lines 631-645.

**Section 4.5**

**additional text：**

(5) The probit-derived preferred PET for the frail group (29.0°C) fell outside the empirical PET range. This value is a statistical extrapolation and should not be interpreted as a valid quantitative estimate. Consequently, we treat it only as a directional indicator (frail > pre-frail > non-frail). Future studies should employ all-season measurements or controlled chamber experiments to obtain reliable absolute preferred temperatures for frail older adults.

This addition appears on Lines 803-808.

**(c) Conclusion section**

**Original text:**

Although the outdoor thermal sensitivity of the older people is low, the preferred temperature is high, indicating that the frail older people want a warmer thermal environment.

**Revised text:**

Frail older adults expressed a stronger preference for warmer conditions compared to pre-frail and non-frail groups, although the absolute preferred PET could not be reliably quantified within the winter measurement range.

This addition appears on Lines 827-829.

By the way, according to the ethics statement requirements from editor, we added a paragraph in the begining of section 2.3 Questionnaire survey, explaining the survey consent from all participants.

We hope these revisions address the reviewer’s concern by removing the claim of quantitative reliability and re-framing the result as a hypothesis-generating directional finding. This addition appears on Lines 235-244.

**Thank you again for helping us improve the scientific rigor of our manuscript.**
